# Effect of Irisin on LIF and integrin αvβ3 in rats of implantation failure

**DOI:** 10.1186/s12958-021-00700-9

**Published:** 2021-02-03

**Authors:** Li Zhou, Chenggang Li, Xiangshu Liu, Tao Zhang

**Affiliations:** 1 Department of Traditional Chinese Medicine, Affliated Dongfeng Hospital, Hubei University of Medicine, Shiyan, Hubei 442000 P.R. China; 2 Hubei Key Laboratory of Wudang Local Chinese Medicine Research, School of Pharmaceutical Sciences, Hubei University of Medicine, Shiyan, Hubei 442000 P.R. China

**Keywords:** Irisin, Endometrium, Leukemia inhibitory factor, Integrin αvβ3

## Abstract

**Objective:**

The aim of this study is to investigate the effect of irisin on leukemia inhibitory factor (LIF) and integrin αvβ3 in implantation failure uterus.

**Methods:**

Early pregnant rats were randomly divided into normal group (N), mifepristone treated group (M), irisin group (I) and progestin group (P). The implantation failure model was established using mifepristone. Second, we evaluated the average number of embryos and detected the expression of LIF and integrin αvβ3 protein and mRNA in endometrium.

**Results:**

Compared with group M, the average number of embryos was significantly higher in group N, P and I, the expression of LIF and integrin αvβ3 in endometrium was significantly higher in group N, P and I.

**Conclusion:**

Irisin could improve the poor receptive state of endometrium by promoting LIF and integrin αvβ3 secretion to improve blastocyst implantation in rats of implantation failure.

## Background

Irisin, a newly discovered myokine, is released from the muscle immediately after exercise. It is secreted from fibronectin type III domain containing 5 (FNDC5) after the cleavage of its extracellular protein [1]. It drives brown-fat-like conversion of white adipose tissues (WAT) and therefore has been suggested to improve metabolic and glucose homeostasis [2]. Since its discovery, irisin has been the subject of many studies due to its physiopathyological role. Irisin has initially been described as a protective factor against diet-induced weight gain, mediated by browning of WAT and thus increased energy expenditure [3]. Many subsequent studies have investigated a potential role in metabolic diseases, including obesity, type 2 diabetes mellitus (T2DM), nonalcoholic fatty liver disease (NAFLD), lipid metabolism and cardiovascular disease (CVD), polycystic ovary syndrome (PCOS), and metabolic bone diseases [4]. However, the reproductive effects of this hormone, if any, remain largely unexplored.

Reproductive capability is closely linked to the metabolism condition of the adipose tissue, both obesity and undernutrition have been found to be associated with reproductive dysfunction [5, 6]. Existing evidence of the role of biological factors, such as leptin, insulin and adiponectin, whose main function is to regulate energy metabolism, in reproduction is conclusive [7, 8]. Moreover, studies have shown that the expression of irisin has been identified in endometrium and ovary [9, 10]. Based on these studies, we speculate that, irisin, a new molecular marker and target in metabolic disorder [11], may have potential effect on reproductive capacity. Due to the fact that receptive endometrium is the key for implantation at the early stage of successful pregnancy [12], the aim of this study was to investigate the effects of irisin on the expression of endometrial receptivity markers, including leukemia inhibitory factor (LIF) and integrin αvβ3 at the implantation sites in implantation failure uterus.

## Materials and methods

### Animals and grouping

Sexually mature female wistar rats (*n* = 64), weight 210-230 g, and reproductive adult male wistar rats (*n* = 32), weight 250-300 g, SPF grade, were provided by the Medical Experimental Animal Center of Hubei Medical College. All rats were adaptively fed for 3 days. Observed the estrogen cycle of female rats, and rutting female rats were mated with male rats at 6 pm and checked the viginal smear at 8 am the next day. The first day of pregnancy (D1) was defined as sperm detection on the vaginal smear. Then randomly divided pregnant rats into normal group (N, *n* = 16) and implantation failure group (*n* = 48).

### Modeling and treatment

The mifepristone tables (Qingdao Jie Shi Kang Biotechnology Co., Ltd. China) is dissolved in appropriate amount of edible sesame oil and the ultimate concentration was 2 mg/ml. Rats in implantation failure group were treated with mifepristone solution at 5.5 mg/kg by neck subcutaneous injection on D1 at 9 am to establish the implantation failure model, while rats in group N were given an equal amount of sesame oil. Then randomly divided implantation failure rats into mifepristone treated group (group M, *n* = 16), irisin group (group I, *n =* 16) and progestin group (group P, *n =* 16).

Then rats in group I and group P were respectively intramscularly injected with recombinant of irisin (Beijing Baiao Laibo Technology Co., Ltd. China; 100 ng/g.d), and progestin (Zhejiang Xianju Pharmaceutical Co., Ltd., China; 40 mg/kg.d) from D1 to D6, while the group M and group N were injected with an equal volume of normal saline at the same time. Then rats in each group were equally randomized into 6-day pregnancy group and 10-day pregnancy group (respectively defined as N6 and N10, M6 and M10, I6 and I10, P6 and P10; *n* = 8 for all groups). Rats in 6-day pregnancy group were sacrificed after injection on the 6th day of pregnancy, while rats in 10-day pregnancy group were raised to the 10th day of pregnancy and sacrificed.

### Tissue processing

Rats were intraperitoneal narcotized with 2% pentobarbital sodium at 4 pm on D6 and D10 for each group. The uteri were collected by laparotomy, the number of fetuses was counted for 10-day pregnant rats. The uteri of 6-day pregnant rats were scissored under anatomical lens. The endometria where the blastocysts attached to were collected, part of the endometrial were fixed in 4% paraformaldehyde, to be paraffin embedded, sliced; while others were stored at − 80 °C for later use.

### Histological parameters

Uterine tissues were fixed and sections of 5 μm thickness were cut and stained with hematoxylin and eosin (HE) according to standard procedures. The morphology of endometrium was observed using Micro Image.

### Immunohistochemistry for LIF and integrin αvβ3

Paraffin sections were kept in oven at 60 °C for 1 h. Then these sections were deparaffinized and rehydrated through degraded ethanol. After that, antigen retrieval was performed by incubating these sections in 0.01 M citrate buffer (PH 6.0) at 98 °C for 20 min. Then, endogenous hydrogen peroxidase activity was quenched using 3% H_2_O_2_. Sections were blocked with 5% bovine serum albumin (BSA) for 30 min. Excess BSA was drained. These sections were then incubated in respective primary antibody overnight at 4 °C. Primary antibodies against LIF and integrin αvβ3 were raised in rabbit (Boster Biological Technology Co., Ltd. China). For the negative control, slides were incubated in PBS. Then these sections were incubated with HRP labeled goat anti-rabbit IgG at 37 °C for 1 h and incubated with substrate diaminobenzidine for 3–5 min and counterstained with Harris hematoxylin. Pictures were taken by a Nikon Micro-imaging system under 40 times light microscope, ten fields per slice, and analyzed with Image-Pro Plus 6.0 to measure average optical intensity (AOI).

### Elisa for LIF and integrin αvβ3

Uterus was chopped and homogenated, extracted total protein and detected protein content using BCA method. Samples were diluted to a concentration of 1-10μg/ml, then take 100ul of sample for Elisa. The procedure of quantitative sandwich enzyme immunoassay technique was followed according to the manufacturer’s protocols (Cusabio Biological Technology Co., Ltd. China).

### Real-time PCR for LIF and integrin αvβ3

Uterus was dissociated with Trizol reagent (Takara Biotechnology Co., Ltd. China) and total RNA was extracted according to the manufacturer’s protocols. RNA purity and concentration were measured using a nucleic acid/protein analyzer. 1μg of extracted total RNA was reverse-transcribed with reverse transcription kit according to the manufacturer’s protocols (Takara Biotechnology Co., Ltd. China) in Mastercycler gradient PCR apparatus. The primers were designed according to published sequences. Then 50ul of reaction system was performed for PCR amplification. Real-time PCR reactions were performed using an Applied Biosystems Step-One Real-Time PCR System, thermal cycler protocol: stage 1, Reps1 95 °C 30 s; stage 2, Reps40 95 °C 5 s, 60 °C 30 s; stage 3, Reps1 95 °C 15 s, 60 °C 1 min, 95 °C 15 s. The data were analyzed by the 2^−ΔΔCT^ method.

Primer sequences:

β-actin.

Forward primer: 5′-GGAGATTACTGCCCTGGCTCCTA-3′.

Reverse primer: 5′-GACTCATCGTACTCCTGCTTGCTG-3′.

LIF

Forward primer: 5′-ATCAAGAGTCAACTGGCTCAACTCA-3′.

Reverse primer: 5′- TGTTGGGCGCACATAGCTTAT-3′.

Integrin αvβ3.

Forward primer: 5′-TTCAATGCCACCTGCCTCAA-3′.

Reverse primer: 5′-TGAAGCTCACCGTGTCTCCAA-3′.

### Statistical analysis

Data were presented as the mean ± standard deviation (SD). Statistical analysis was performed using SPSS 16.0 statistical software. Differences between any two groups were analyzed by independent t-test, and one-way analysis of variance (ANOVA) was used in a multigroup comparision, Chi-square test was used to compare qualitative data between groups. All *p*-values < 0.05 were considered statistically significant.

## Results

### Embryo implantation in rat uteri

**Fig. 1:**
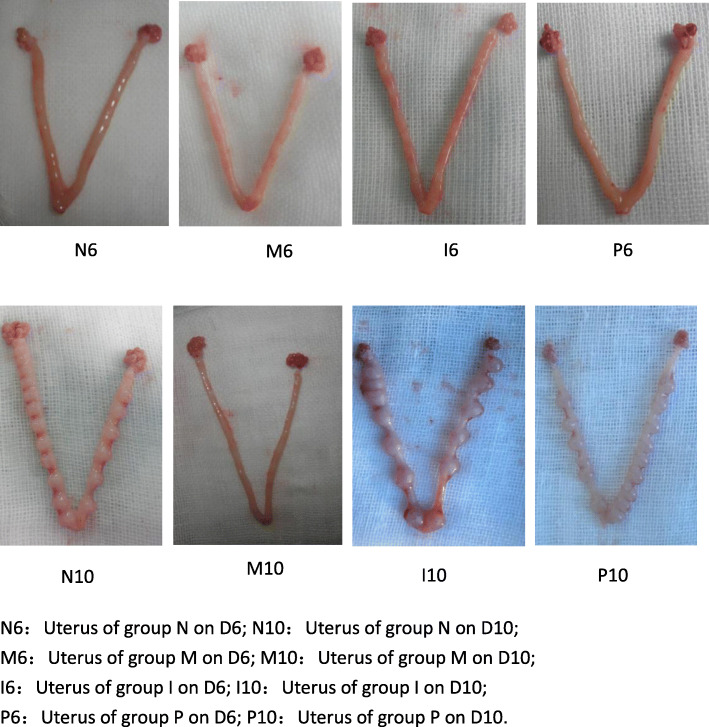
Appearance of rat uterus

**Fig. 2:**
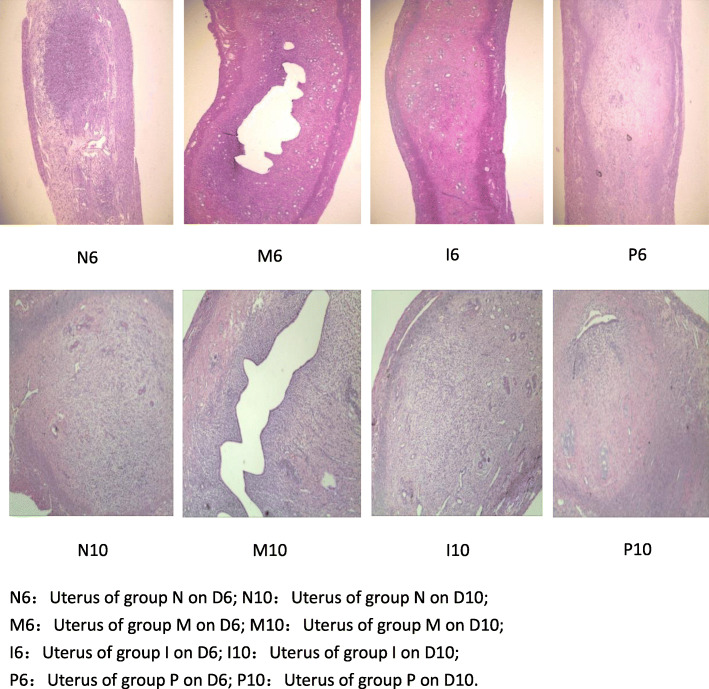
Morphological changes observed under light microscope (Original magnification: × 40)

The uteri were examined for the number of implanted embryos and the morphological status. (Table 1, Figs. 1, 2).

**Table 1:** Pregnancy rate and average number of implanted embryos(±S)

Groups	Pregnancy rate	Average number of implanted embryos
N(*n =* 8)	(7)87.5%	14.64 ± 4.60
M(*n =* 8)	(2)25%^a^	2.71 ± 1.25^a^
I(*n =* 8)	(7)87.5^b^	13.76 ± 5.31^b^
P(*n =* 8)	(8)100%^b^	13.86 ± 4.75^b^

^a^represents that there is significant difference when group M is compared with group N (*P* < 0.05)

^b^represents that there is significant difference when group I and P is compared with group M (*P* < 0.05)

Uterus in group M is pale, the distribution of implantation sites is asymmetric, the size of embryos is smaller and the uterine cavity is not closed. In contrast, uterus in group N, group P and group I is bright red, the distribution of implantation sites is symmetric, the embryos is replete and the uterine cavity is closed.

The average number of implanted embryos in group M is significantly decreased than that in group N (*p* = 0.01), however, the average number of embryos in group I and group P is obviously increased when compared with group M (*p* = 0.002, *p* = 0.001, respectively). No significant differences were found in group P, group N and group I (*p* = 0.931).

### Outcome of Elisa

Endometrial LIF and integrin αvβ3 protein level on D6 is significantly lower in group M then that in group N (both *p* < 0.001), while the level of LIF and integrin αvβ3 protein in group I and group P is obviously increased when compared with group M (*p <* 0.001 for both). No significant differences were found in group P, group N and group I (LIF: *p* = 0.144; integrin αvβ3: *p* = 0.229). (Table 2).

**Table 2:** Comparison of endometrium cytokines protein concentration by Elisa.(±S)

Groups(*n =* 8)	LIF (pg/ml)	integrin αvβ3(pg/ml)
N	72.96 ± 8.13	83.98 ± 7.62
M	20.14 ± 2.88^a^	36.61 ± 5.47^a^
I	66.87 ± 6.89^b^	87.32 ± 8.98^b^
P	71.47 ± 7.73^b^	79.14 ± 6.38^b^

^a^represents that there is significant difference when group M is compared with group N (*P* < 0.001)

^b^represents that there is significant difference when group I and P is compared with group M (*P* < 0.001)

### Outcome of immunohistochemistry

**Fig. 3:**
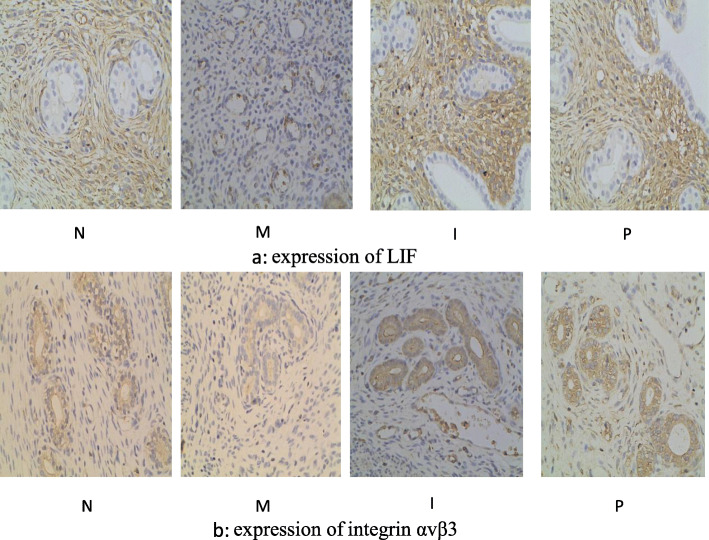
Immunohistochemical staining (Original magnification: × 400)

As is shown in Fig. 3, endometrial LIF protein is mainly expressed in mesenchymal cells, while integrin αvβ3 protein is mainly expressed in luminal and glandular epithelium. The AOI of LIF and integrin αvβ3 in group M is significantly decreased then that in group N (both *p* < 0.001); when compared with group M, the AOI of LIF and integrin αvβ3 is obviously increased in group I and group P(*p* < 0.001 for both). No significant differences were found in group P, group N and group I (LIF: *p* = 0.090; integrin αvβ3:*p* = 0.297). (Table 3, Fig. 3).

**Table 3:** Comparison of endometrium cytokines protein average optical intensity by immunohistochemistry.(±S)

Groups(*n =* 8)	Average optical density	
	LIF	integrin αvβ3
N	0.42 ± 0.09	0.47 ± 0.12
M	0.13 ± 0.05^a^	0.16 ± 0.05^a^
I	0.36 ± 0.07^b^	0.41 ± 0.11^b^
P	0.43 ± 0.11^b^	0.39 ± 0.12^b^

^a^represents that there is significant difference when group M is compared with group N (*P <* 0.001)

^b^represents that there is significant difference when group I and P is compared with group M (*P <* 0.001)

### Outcome of RT-PCR

Compared with group N, endometrial LIF and integrin αvβ3 mRNA is significantly decreased in group M (both *p* < 0.001); while compared with group M, there is a significantly elevation of the expression of LIF and integrin αvβ3 mRNA in group I and group P(*p <* 0.001 for both). No significant differences were found in group P, group N and group I (LIF: *p* = 0.090; integrin αvβ3: *p* = 0.297). (Table 4).

**Table 4:** Comparison of endometrium cytokines mRNA concentration by RT-PCR(±S)

Groups(*n =* 8)	LIF	integrin αvβ3
N	0.93 ± 0.24	0.78 ± 0.22
M	0.37 ± 0.10^a^	0.31 ± 0.10^a^
I	0.86 ± 0.21^b^	0.73 ± 0.22^b^
P	0.89 ± 0.17^b^	0.81 ± 0.19^b^

^a^represents that there is significant difference when group M is compared with group N (*P <* 0.001)

^b^represents that there is significant difference when group I and P is compared with group M (*P <* 0.001)

## Discussion

Implantation is one of the most essential processes in reproduction and is the key for successful pregnancy. In this process, both energetic blastocyst and receptive endometrium are two prerequisites for successful implantation. Endometrial receptivity refers to a state that endometrial epithelial cells are structurally and functionally prepared for embryo implantation. A number of molecules have been recognized as markers of endometrial receptivity, including ovarian hormones and their receptors, integrin αvβ3, LIF and so on.

It’s known that mifepristone is a classical antagonist to progesterone which specifically blocks the action of progesterone and resists implantation [13]. These well-known endometrial receptivity markers, such as LIF, integrin αvβ3, have the same characteristic, that is, they can all be regulated by progesterone directly or indirectly [14]. Therefore, mifepristone can destroy the endometrial receptivity and inhibit blastocyst implantation. Yet in our study, mifepristone significantly reduced the number of implantation blastocysts and lead to a decrease in endometrial LIF and integrin αvβ3levels, this suggests that we have successfully used mifepristone to establish the implantation failure model, which provides a basis for the futher observation of the function of irisin on the endometrium.

Irisin has gained a marked insight in the field of medical biology and its potential therapeutic importance in metabolic diseases. Even though, it is important to explore the role of irisin in other pathological/physiological conditions.

Studies demonstrated that the expression of irisin has been found in endometrium and endometrial irisin level in PCOS patients decreased [9, 10], which indicated a potential direct role of irisin on endometrium. Embryo implantation is the premise of successful pregnancy, it is crucial to take timely modifications for endometrium to become receptive to the developing embryo for success of implantation [12]. As a result, observing the effect of irisin on endometrial receptivity is of great significance for explore its reproductive effects.

The present study demonstrated that the average number of implantation blastocysts was notably increased in irisin group than mifepristone group, and irisin can improve the reduction of LIF and integrin αvβ3 caused by mifepristone, which confirmed a potential function of irisin on endometrial receptivity. To our knowledge, few studies have reported on the effect of irisin on endometrial function. Our novel finding provides significant information for exploring the reproductive role of Irisin, at the same time provides a new target for the diagnosis and treatment of infertility. However, no exact evidence is available on the possible molecular mechanism of this role in endometrium. Possible explanation of the present study on potential pathways is that irisin regulate energy metabolism in the body and uterus.

Reproductive and fertility are closely linked to energy metabolism and the endocrine function of the adipose tissue [5, 6]. Moderate physical activity is beneficial for reproductive function [15]. Studies have highlighted the endometrium’s role in the pathophysiology of adverse reproductive outcomes among obese women [16–18]. To be more specific, Bellver et al. published a clinical study of 9587 first cycles of ovum donation and showed that implantation, pregnancy, and live birth were significantly reduced as BMI (body mass index) increased, it was suggested that female obesity impairs the reproductive outcome of ovum donation probably as a result of reduced receptivity [19]. Bellver et al. also published a review supporting a role for endometrium in the pathophysiology of reproduction in obese women [20]. Moreover, it has been reported that obesity and exposure to a high-fat diet impair endometrial stromal cell decidualization, which is necessary for uterine receptivity [21]. In addition, studies have highlighted that high concentration of insulin could inhibit expression of endometrial receptivity markers such as integrins, osteopontin [22, 23]. It’s believed that, efficient nutrients exchange between the uteri and fetus, including glycogen, proteins and lipids, is essential for a successful pregnancy [24]. During the early stage of implantation, the embryo is nourished via nutritive endometrial secretions, which derived from endometrial glands, and the secretions play an important role in regulating endometrial receptivity [24–26]. In addition, hormones whose main function is to regulate energy metabolism, such as leptin, is involved in reproductive function and, more precisely, in the embryonic-maternal cross-talk at the time of implantation [27]. Based on these studies, it is reasonable to speculate that, irisin, a hormone whose main function is to regulate energy metabolism and improve insulin resistance, may play a crucial role in endometrial receptivity by regulating systemic metabolism and local nutritional metabolism of the uterus. Our study indicates that irisin may play a major role in metabolic diseases complicated with infertility, such as obesity and PCOS, it can improve endometrial receptivity while regulating metabolic abnormalities, which is superior to progesterone only to improve the function of the endometrium. Our study provides a new perspective for the treatment of obesity, PCOS patients complicated with infertility.

In addition, physical activity has been proposed to provide benefits in women attempting pregnancy, especially those with higher BMI, but the specific mechanism is confusing [15]. since irisin increases energy metabolism and improves the function of the endometrium, we can make a bold hypothesis, that is, physical activity can effectively improve the body’s metabolism and reproductive disorders after increasing the release of irisin. This may provides a scientific basis for women to improve reproductive function through physical activity especially those with higher BMI.

## Conclusions

The present study demonstrated that irisin could improve the poor receptive state in implantation failure uterus by promoting LIF and integrin αvβ3 secretion to improve blastocyst implantation. Future studies to confirm our initial observations and to explore the possible pathways are warranted.

## Data Availability

The datasets used and analyzed during the current study are available from the corresponding author on reasonable request.
